# Insular cavernous malformation resection through a minipterional, transsylvian approach

**DOI:** 10.3171/2019.7.FocusVid.19148

**Published:** 2019-07-01

**Authors:** Peyton L. Nisson, Robert T. Wicks, Xiaochun Zhao, Whitney S. James, David Xu, Peter Nakaji

**Affiliations:** 1 College of Medicine, University of Arizona, Tucson; 2 Department of Neurosurgery, Barrow Neurological Institute, St. Joseph’s Hospital and Medical Center, Phoenix; and; 3 High Desert Surgery Center, Prescott, Arizona

**Keywords:** insula, cavernous malformation, transsylvian, minimally invasive, minipterional, video

## Abstract

Cavernous malformations of the brain are low-flow vascular lesions that have a propensity to hemorrhage. Extensive surgical approaches are often required for operative cure of deep-seated lesions. A 23-year-old female presented with a cavernous malformation of the left posterior insula with surrounding hematoma measuring up to 3 cm. A minimally invasive (mini-)pterional craniotomy with a transsylvian approach was selected. Endoscopic assistance was utilized to confirm complete resection of the lesion. The minipterional craniotomy is a minimally invasive approach that provides optimal exposure for sylvian fissure dissection and resection of many temporal and insular lesions.

The video can be found here: https://youtu.be/9z6_EhU6lxs.

## Transcript

This video illustrates a minimally invasive, endoscopic pterional craniotomy for transsylvian resection of a cavernous malformation. The patient is a 23-year-old female who presented with a single seizure, 1 week of headache, and 1 month of decreased hearing on the left. The MRI scans illustrate a relatively deep-seated cavernous malformation located in the low posterior insula. A traditional approach would involve a pterional craniotomy and transsylvian dissection.^1,6^ We chose a smaller approach, which is a variant on this more classic approach.^2–4^ The patient is positioned supine with Mayfield three-pin fixation, with a small curvilinear incision centered over the pterion slightly on the higher side to allow a trajectory looking down. There is no need to carry this dissection down to the zygoma or more frontally as exposure of more frontal or temporal lobe will not improve exposure of the cavernous malformation. The size of the craniotomy is evident, which is about 2 cm × 2 cm. The dura is flapped up, and the sylvian fissure is exposed. We begin by opening the arachnoid in the classic fashion. We see that there are a number of bridging veins in this area as well as the middle cerebral artery and branches. Careful microdissection is carried out to separate all of these small branches. Gradual dissection is carried down on either side of the middle cerebral artery, making a small corridor that is checked frequently against image guidance. The opening of the sylvian fissure is gradually expanded using sharp microscissor dissection. We make our way deeper into the sylvian fissure. As we go through the deeper layers of arachnoid, we come down onto a small area of brain, which appears somewhat yellow and hemosiderin stained. The image guidance is again checked and reveals a location in the posterior insula that corresponds to the thinnest point. It is not readily apparent at the surface. A very small corticotomy is made, about 5 or 6 mm long, in this location, using bipolar. The opening is gradually expanded using the 5-French suction and the bipolar. Initially we transit a small cap of normal brain. Gradual expansion of this corridor reveals the cavernous malformation. As we come onto the surface, small microdissectors are used to define its walls and separate it from the surrounding brain. Although the cavernous malformation is relatively large, it is possible to move around it by changing trajectories from time to time, and continuously dissecting it away from the gliotic capsule of brain around it. The opening into this zone still is never expanded by more than an additional millimeter or two. Gradually the cavernous malformation is mobilized so that it can begin to roll within its cavity. Here we see further dissection and expansion in each of several directions as we gradually free it from the surrounding brain. Small micropickups with teeth are useful for grasping the cavernous malformation, not to pull it out, but to mobilize it within the cavity. It gradually becomes more mobile as it is manipulated. The cavernous malformation begins to deflate somewhat and becomes increasingly mobile to the point of where it is possible to draw some of it out of the capsule. We deflate the cavernous malformation and drain some of its interior contents, which includes blood of various ages. This allows further mobilization of its thickened wall, which is gradually separated and is drawn up out of the cavity. The cavity is carefully inspected. We can see that the opening is smaller than a half × half [cm] blue-tail patty. An endoscope is then introduced into the cavity to inspect the walls for any residual. Although it is possible to see into the cavity from above, getting this kind of angled perspective, given the very small craniotomy and sylvian fissure opening, would not otherwise be possible. We were able to remove hemostatic agent and look for any sign of solid cavernous malformation. None was seen. The patient did well afterwards and was neurologically intact with no language deficits and with cessation of her seizures.^5^

### Time points

0:38 Preoperative MRI
0:57 Illustration of minipterional approach
1:20 Intraoperative size of the minipterional craniotomy
1:29 Sylvian fissure dissection
2:11 Opening of the insula
2:56 Mobilization of the cavernous malformation
4:17 Deflation of the cavernous malformation interior contents
4:24 Drawing cavernous malformation out of the cavity
4:46 Introduction of a rigid endoscope for inspection of the cavernous malformation cavity
5:10 Postoperative MRI

## References

[1] Altay T Couldwell WT The frontotemporal (pterional) approach: an historical perspective Neurosurgery 71 481 492 2012 22472552 10.1227/NEU.0b013e318256c25a

[2] Caplan JM Papadimitriou K Yang W Colby GP Coon AL Olivi A The minipterional craniotomy for anterior circulation aneurysms: initial experience with 72 patients Neurosurgery 10 Suppl 2 200 207 2014 24625424 10.1227/NEU.0000000000000348

[3] Figueiredo EG Deshmukh P Nakaji P Crusius MU Crawford N Spetzler RF The minipterional craniotomy: technical description and anatomic assessment Neurosurgery 61 256 265 2007 10.1227/01.neu.0000303978.11752.45 18091240

[4] Hamby WB Pterional approach to the orbits for decompression or tumor removal J Neurosurg 21 15 18 1964 14110353 10.3171/jns.1964.21.1.0015

[5] Taslimi S Ku JC Modabbernia A Macdonald RL Hemorrhage, seizures, and dynamic changes of familial versus non-familial cavernous malformation: systematic review and meta-analysis World Neurosurg 126 241 246 2019 30851471 10.1016/j.wneu.2019.02.115

[6] Tirakotai W Sure U Benes L Krischek B Bien S Bertalanffy H Image-guided transsylvian, transinsular approach for insular cavernous angiomas Neurosurgery 53 1299 1305 2003 14633296 10.1227/01.neu.0000093496.61236.66

